# Flexibility in Problem Solving and Tool Use of Kea and New Caledonian Crows in a Multi Access Box Paradigm

**DOI:** 10.1371/journal.pone.0020231

**Published:** 2011-06-08

**Authors:** Alice M. I. Auersperg, Auguste M. P. von Bayern, Gyula K. Gajdon, Ludwig Huber, Alex Kacelnik

**Affiliations:** 1 Department of Cognitive Biology, University of Vienna, Vienna, Austria; 2 Konrad Lorenz Institute for Ethology, Vienna, Austria; 3 Department of Zoology, University of Oxford, Oxford, United Kingdom; Duke University, United States of America

## Abstract

Parrots and corvids show outstanding innovative and flexible behaviour. In particular, kea and New Caledonian crows are often singled out as being exceptionally sophisticated in physical cognition, so that comparing them in this respect is particularly interesting. However, comparing cognitive mechanisms among species requires consideration of non-cognitive behavioural propensities and morphological characteristics evolved from different ancestry and adapted to fit different ecological niches. We used a novel experimental approach based on a Multi-Access-Box (MAB). Food could be extracted by four different techniques, two of them involving tools. Initially all four options were available to the subjects. Once they reached criterion for mastering one option, this task was blocked, until the subjects became proficient in another solution. The exploratory behaviour differed considerably. Only one (of six) kea and one (of five) NCC mastered all four options, including a first report of innovative stick tool use in kea. The crows were more efficient in using the stick tool, the kea the ball tool. The kea were haptically more explorative than the NCC, discovered two or three solutions within the first ten trials (against a mean of 0.75 discoveries by the crows) and switched more quickly to new solutions when the previous one was blocked. Differences in exploration technique, neophobia and object manipulation are likely to explain differential performance across the set of tasks. Our study further underlines the need to use a diversity of tasks when comparing cognitive traits between members of different species. Extension of a similar method to other taxa could help developing a comparative cognition research program.

## Introduction

Cognitive mechanisms evolve to a large extent in response to selective pressures peculiar to each species’ ecology, physiology and morphology [1]. Because of this, cognitive processes may differ qualitatively and quantitatively across species.

One important tool of comparative cognition is to analyze the performance of different species facing the same task. However, if two species are to be compared on the basis of their final performance on a single task then task-specific factors may lead to differences that are not indicative of general problem-solving ability but are instead expressing different motivational predispositions or other non-cognitive specialisations.

A well-argued response to this problem is using a battery of different tasks rather than a single one [2]–[6]. It is also essential to use a sufficiently diverse set of tasks. For instance, comparing tool-using and non-tool using species only in tool-using tasks and reaching conclusions in terms of problem-solving ability would make little sense, as would using only tasks that favour proactive rather than passive solutions.

We then start from the premise that focusing on species-specific mechanisms and on the strategies that underlie problem-solving performance in a diversity of contexts is more informative than attempts to rank individuals or species on problem-solving success without due reflection on the cognitive demands of the tasks employed. This logic could be followed by offering several tasks simultaneously and by successively removing the options that have already been mastered. A survey of discrepancies between species of which tasks are approached first, how many solutions are discovered, how quickly the species re-learn, overcome habits and adapt to changes by switching between different solutions, may expose factors influencing species-specific traits such as behavioural flexibility, neophilia, exploration strategy, attention, motivation, affordance learning, anatomical constraints and other traits of interest. Apparatuses offering different physical problems at the same time have been used in various experiments targeting intra-specific social learning (rather than technical competence) eg. [7]–[10], but to our knowledge, have not been used for inter-specific comparisons of the mechanisms underlying problem solving.

We applied this approach to two highly competent extractive foragers: a parrot (the kea, *Nestor notabilis*) and a corvid (the New Caledonian crow, *Corvus moneduloides*). These birds are both representatives of two avian families that stand out for having larger encephalization quotients [11]–[13] and greater innovation scores [14]–[16] than other avian taxa, and that seem to parallel the great apes in performance in some physical tasks [17]–[26]. Kea are neophilic mountain parrots of New Zealand with well-known manipulative skills that are not known to use tools in the wild, while New Caledonian crows are known for their tool manufacture and use in the wild [27]–[29]. Both species are renowned for their problem solving skills in various physical cognition tasks [8], [17], [18], [20], [23]–[26]; [30]–[32]. Both species are generalists that live in social groups and within complex environments with fluctuating resources and pursue a food extracting foraging style, kea digging for roots in the ground of mountain plains and crows fishing for larvae in decaying tree logs [27], [29], [33]. Our captive kea had also shown competence in the use of compact objects, such as wooden blocks, as tools to access rewards [18], [30]. This gives us an opportunity to disentangle various possible cognitive and non-cognitive factors influencing differential performance in problem solving in a natural tool user as well as a naturally non-tool using species.

One of the issues that motivate our study concerns the evolution of tool use and its cognitive underpinnings, in particular whether there is something special about the cognition associated with tool use and other forms of complex object manipulation. For example, it is possible that adaptive specialization to a given type of tool use may either enhance or impair performance in other tool-related or object-manipulation tasks. It has been argued, that while tool-related behavior may not necessarily be more cognitively demanding than other forms of problem-solving, it may be more revealing of the information-processing that it involves, and hence may be useful to expose what animals understand about the relationship between objects and the effects objects have on one another [33]. So far researchers have attempted to experimentally determine the role of factors such as pre-functional development [28], [34] (associative) experience, affordance learning [24], self-control, planning and reasoning about invisible forces [35]. Here we focus on how the specificity of tasks determines differential performance in a comparative setting.

We developed a “Multi Access Box” (MAB), as a tool to compare problem solving in extractive foraging species. The MAB features a battery of tasks that all lead to the same goal, a food reward presented in the centre of a transparent box. Initially the subject is allowed to choose any of the four options, but once a subject has developed a consistently successful performance with each technique to access the food, this solution is blocked and we record its performance in establishing competence in an alternative strategy. Six kea and five crows were exposed to this setup, where two of the tasks required the use of a mediating object as a tool (either a stick or a ball). The other two solutions did not require tool use and involved either pulling a string tied around the reward or pulling from a hook handle to open a window.

## Results

### First Session

**Figure 1:**
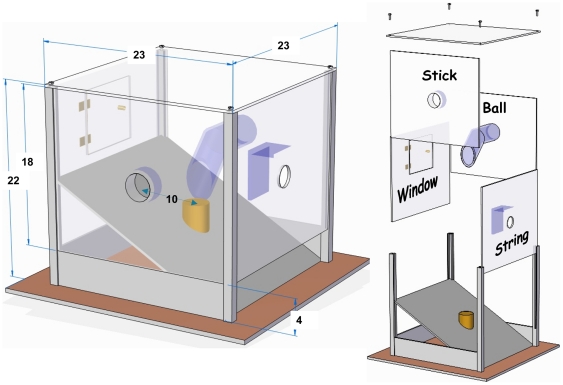
The Multi-Access-Box (MAB). Notice the four exchangeable transparent walls with openings corresponding to the 4 possible solutions (string, window, ball and stick). Dimensions in cm.

**Figure 2:**
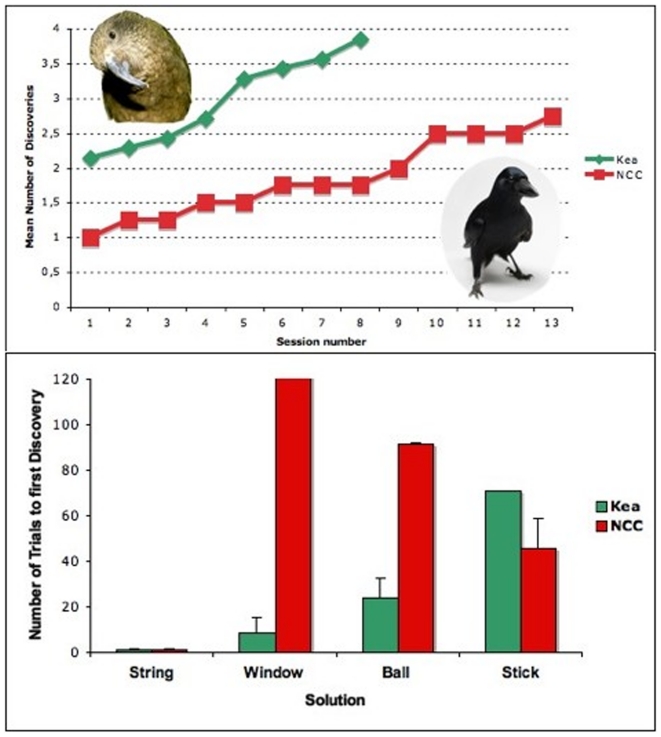
First discoveries of the various solutions. A) Mean number of first discoveries throughout sessions (the last discovery was the window solution by the NCC Uek in session 13). Kea are represented by the green line; NCC by the red. B) Mean number of trials until a solution was discovered. Green  =  kea; red  =  NCC. T-bars represent SE.

There were differences in the way kea and crows behaved towards the apparatus (see apparatus in Figure 1), and this translated in differences in the number of solutions discovered (namely used successfully at least once) within the first session. Averaging across individuals, NCCs found 0.75 (Range 0–1) and kea 2.33 (Range 2–3) solutions within the first session (see Figure 2). Also within the first session, all kea touched (i.e. made physical contact with) all four opening devices and both tool types, while the NCCs rarely touched the apparatus (except the string). The crow Annie-Claude was excluded from testing after failing to retrieve the food reward within the time given during the habituation phase.

### Entrance Manipulations

The kea directly manipulated the opening devices more often than the crows did in the course of this study: When repeated touchings are considered, the mean percentages of trials in which five or more manipulations of an entrance occurred were 18% +/−1.92 SE, N = 6 in the kea than in the crows (Mann-Whitney U test; Z = 2.34; p = 0.01). Entrance manipulations by the crows consisted almost exclusively of brief pecking actions at the openings with the beak tip or with a tool, while the kea’s manipulations included violent pulling and tearing as well as rocking, probing, scratching, and levering of the physical parts of the apparatus.

### Sequence of solutions discovered and established

After an initial period distributing their attention broadly around the apparatus, all subjects, kea and crows, focussed on the string option. After the string solution was blocked, the subsequent order of solutions established differed between the two species and, in kea, between individuals (see Table 1).

**Table 1:** Order and session in which each individual reached criterion (8 consecutive times correct or 9 out of 10 correct) for each of the four solutions (string, window, ball and stick).

*Species*	*Ind*	*1^st^solution*	*Session*	*2^nd^solution*	*Session*	*3^rd^solution*	*Session*	*4^th^solution*	*Session*
**Kea**	Br	String	3	Window	4	Ball	6	-	-
	Fr	String	2	Window	3	Ball	5	-	-
	Ke	String	2	Ball	4	Window	5	Stick	10
	Lu	String	2	Window	4	Ball	5	-	-
	Pi	String	3	Window	4	Ball	6	-	-
	Ta	String	3	Ball	4	Window	6	-	-
**Average**			2.5		3.83		5.5		10
**NCC**	Bk	String	2	-	-	-	-	-	-
	Ey	String	2	Stick	8	Ball	13	-	-
	Ti	String	1	Stick	12	-	-	-	-
	Uék	String	2	Stick	4	Ball	8	Window	12
**Average**			1.75		8		10.5		12

Three NCCs next reached criterion using the stick tools (between session 4 and 12) and, when this option was blocked, two of these reached criterion with the ball tool (in sessions 8 & 13; see Table 1). Finally, one of these two crows (Uék) solved the window hook task. In many cases the crows touched the window (which could not be opened that way) with a stick, rather than trying to pull the hook. On average 31.77% +/−8.00 SE, N = 3 of the ineffective tool actions (ITA =  inserting or bringing the tool into contact with an inappropriate opening; note that in an ITA a tool has to touch an opening and not just any part of the apparatus) in the trials in which the crows finally used a tool to retrieve the reward were directed against the window option. In fact, after learning to open the window by pulling from the hook, Uék always reached for a stick and used it to poke the reward out, instead of sticking her head through the window and taking the reward directly, as the kea did (see Movie S2).

The kea met criterion in either the ‘ball’ or the ‘window’ option after the string solution was blocked (all kea finished these three solutions). After these options were closed as well, all attempted to use a stick tool, touching the box several times at the appropriate side with the stick (i.e. carrying the tool to the correct opening and touching it with the tool), but failed to insert it. Only one kea, Kermit, succeeded in developing a successful technique to retrieve the reward with the stick. Due to the curvature of their beaks, kea cannot hold a stick in alignment with their heads to the same degree as New Caledonian crows and as a consequence have less control over the tip of the tool. Kermit developed a special routine. He (1) took the tool end *laterally* into his beak and pushed that end into or against the tool entrance. (2) He then switched from grabbing the stick with the beak to grabbing it with the foot, continuing to press the tool end against the opening, (3) Meanwhile he shifted the beak to the end of the tool that was distal to the opening securing the tool’s position with the foot at the tool entrance. Finally (4) he directed the tool through the opening with his beak and manoeuvred it until it hit the reward (see Movies S1, S2). Kermit successfully used the stick tool for the first time three sessions after all other openings had been closed (in session eight) and reached criterion in session ten.

### Speed in switching from one solution to the next

The kea tended to be faster than the NCCs at meeting criterion in new solutions once the previous method was blocked. They took less sessions on average (1.3 +/−0.21 SE, N = 6) to switch from the first to the second option than the three crows that did master a second option (which took 4, 11 and 2 sessions; Mann-Whitney U test; Z = 2.19; p = 0.048). The kea took on average 1.67 +/−0.21 SE, N = 6 sessions to switch from option 2 to option 3 while the two crows that mastered a third option, took 4 and 5 sessions respectively (Mann-Whitney U test; Z = 2.145; p = 0.071). Only one subject of each species mastered a fourth option. The crow took 4 sessions to reach criterion from the ball (3^rd^ option) to the window (4^th^ option), while the kea took 5 sessions to reach criterion from the ball option (3^rd^ option) to the stick (4^th^ option; see Table 1). Establishing the stick solution after the string solution had been blocked, took the three successful crows 5.8 sessions on average even though the task taps into their proven skills as natural tool users (Table 1 & Figure 2).

### Tool preference and other analyses related to the tool solutions

The crows only successfully used the thin sticks and the single successful kea used only the thick sticks. Both species used both sizes of ball tools.

After the string opening had been blocked, the mean number of times the kea brought a ball tool into contact (by touch or insertion) with the apparatus’s openings was significantly higher than with a stick (Wilcoxon signed-rank; T = 2.02; p = 0.043). After the ball option was blocked, balls were removed after three unsuccessful sessions in which all options except for the stick option were blocked for 5 of the 6 kea, because they continued to make attempts to use these now non-functional objects as tools. Nevertheless, no additional kea managed to reach the food with a stick. For the crows the reverse situation was true; they tended to touch openings with the sticks more often than with the balls. However, this was only significant at the 6% level (Wilcoxon signed-rank; T = 1.87; p = 0.06).

The three crows that did reach criterion using tools tried to insert the stick more often into openings than the six kea (Mann-Whitney U test; Z = 2.59; p = 0.01) whilst the kea combined the ball more often than the crows (Mann-Whitney U test; Z = 1.9; p = 0.05).

**Figure 3:**
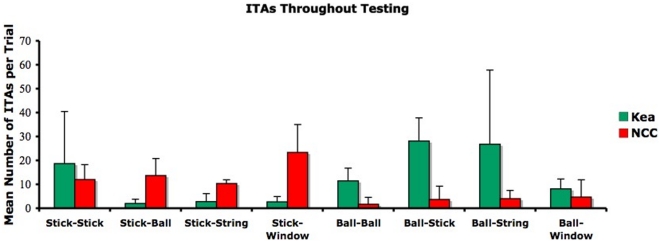
Mean (± SD) number of Ineffective Tool Actions (ITA)/trial throughout all trials in which performance was not immediately successful (excluding data in which of the two tools was removed); green bars  = kea, red bars  =  NCCs; e.g. Stick-Ball indicates the mean number of times the stick was brought in contact with the ball entrance per trial (in which performance was not immediately successful).

We find similar preferences looking only at trials in which ineffective tool actions occurred (see Figure 3). The kea brought the ball more often into contact with an inappropriate opening (excluding trials in which the performance was immediately successful) than the NCCs (Mann-Whitney U test; Z = 2.32; p = 0.024).

In trials including ITAs, the kea also inserted the ball into openings such as the string or the stick opening rather than the window, while the NCCs also touched the window opening frequently with a stick tool (see Figure 3). In incorrect tool use trials, in which the animals conducted ineffective ITA before succeeding to insert the tool appropriately, the average number of ITA per trial was similar in crows and kea (see Table 2).

**Table 2:** The frequency of Ineffective Tool Actions (ITA) in trials in which either the ball or the stick was used to retrieve the reward.

Species	*subjects*	*Ball* *Mean No.* *ITA/trial*	*% ITA* *Stick*	*% ITA* *Ball*	*Stick* *Mean No.* *ITA/trial*	*% ITA* *stick*	*% ITA* *Ball*
**Kea**	Fr	1.38	0	100			
	Br	1.27	17.85	82.14			
	Ke	0.61	23.07	76.92	0.45	33.33	66.67
	Lu	0.4	0	100			
	Pi	1.33	10.71	89.28			
	Ta	0.58	14.28	85.71			
**NCC**	Ey	1.06	79.41	20.59	0.52	100	0
	Ti				0.75	100	0
	Uék	1.41	61.29	38.71	0.357	100	0
**Mean**	**Kea**	**0.93**	**10.98**	**89,01**	**0.45**	**33.33**	**66.67**
	**NCC**	**1.23**	**70.35**	**53,09**	**0.54**	**100**	**0**

This table depicts the mean number of ITA per trial in which the reward was finally retrieved using the ball tool (column 3) or the Stick tool (column 6) as well as the % of ITA in which the stick (or the ball respectively) was inserted into inappropriate openings before succeeding with one of the two tools.

## Discussion

At least one individual of each species discovered all four available methods. This proves that in principle, the affordances of the tasks lay within the cognitive and physical capacity of both species.

There were, however, interesting differences in performance with the present set of tasks. The kea were faster in discovering multiple solutions and showed more individual variation than the naturally tool-using NCCs. Within the first session, all kea successfully employed at least two or three solutions while none of the crows used more than one. The kea also switched to other solutions quicker once previously mastered solutions were blocked. Although both species had experience with compact objects [18], [24], [30], using the ball was acquired faster by the kea, while in the stick option the naturally stick-tool using NCCs were faster. Only one kea succeeded in inserting the stick tool into the correct opening, although all attempted to do so.

To a large extent, differences in exploration patterns and affordance learning as well the balance between neophilia/neophobia seem to be responsible for the differential performance. The kea showed more haptic exploration while the crows, probably due to their higher level of neophobia, seemed to explore more in a visually guided manner. Similar differences exist between kea and common ravens [36]. The kea’s higher readiness to manipulate i.e. act on novel objects, may help them to detect functional affordances. In their naturally low-risk, variable environment, neophilia may reflect low predation risk [20], [37]. The crows, in contrast, approached the apparatus hesitantly, and touched it less often than the kea. Two crows never explored the box thoroughly with their beaks. One of them had to be excluded from the study because it never approached the experimental setup. Neophobia hampered the crows’ performance in other respects. For instance, despite their predisposition, experience and competence for stick tool use, the crows did not use the available tools as first option and also took some time to use tools after the string pulling option had been removed. (The string option was the first to be blocked for both species. It may have represented one of the more conspicuous affordances of the apparatus or evoked little neophobia since both species were familiar with strings; see methods). It is possible that because the available sticks were smoother, painted, and considerably thicker than those the crows naturally choose in the wild [38] ergonomic difficulties synergised with neophobia, and may have affected their performance. Once the crows had established stick tool use, they continued to use the sticks for exploring the box instead of touching it directly. This is reflected in the number of ITA with sticks when confronted with the ball and window option, i.e. after the stick option was blocked. This may indicate a preference to explore unfamiliar objects with tools rather than touching them directly, as shown by Wimpenny et al. (2010) [39]. Such behaviour is, however, disadvantageous in situations where the functionalities of the objects are not detectable by simply applying pressure, such as in the case of the window option, illustrating one cost of the otherwise useful capacity to use tools for exploratory goals.

Another strong difference was that the kea showed greater destructiveness than the crows, as a consequence of their forceful and frequent use of pulling and tearing actions. One kea, Luke, even broke the Plexiglas® on the top of the box while trying to force it open, and most kea attempted to turn over the MAB, which had to be fixed to the aviary floor. Wild kea are well-known for what human observers usually describe as curiosity, playfulness and urge to tear apart objects such as cars’ windshield wipers or picnic baskets [27].

Wild (as well as naïve captive) NCCs also use tearing actions when manufacturing tools from pandanus leaves [29], [40]. They however did not use such behaviours in this setup. We can at this point speculate that tearing behaviours may mainly be orientated towards tool making and nest building rather than to exploration of novel objects or food extraction. The slight curvature at the beak tip of most corvids has been interpreted as an adaptation to feeding on carcasses [41]. NCCs might have secondarily lost their beak curvature making their beaks less suited for violent tearing actions. Very recently Rutz et al. (2010) [42] were able to show that a substantial amount of the crows’ protein and lipid intake came from wood boring beetle larvae obtained with stick tools, also indicating that explorative foraging is more concentrated on probing actions. It seems therefore possible that the range of exploration techniques during foraging in NCC may be constrained by the adaptive specialization for tool use and that this will affect the “zone of latent solutions” [43] within the species' cognitive repertoire.

Our results also provide the first experimental evidence of stick tool use in a parrot. Kea are neither natural tool users like New Caledonian crows, nor do they construct nests with twigs as all corvids do, thus lacking the predisposition of nest builders for handling twigs and other elongated stick-like objects [44]. Instead, kea use or dig burrows for laying their eggs [45]. Also importantly, ergonomically, the use of sticks is clearly difficult for kea. The curvature of their beaks and pronounced size difference between upper and lower beak, precludes a good grip and control of long, straight tools. NCCs, maybe as an adaptation to tool use [41], have short straight beaks with the mandible almost as long as the maxilla, allowing them to effectively hold sticks directly forwards, functionally elongating their beaks and increasing their reach.

To overcome these difficulties, the single successful individual kea developed a complicated stepwise technique, involving carefully concerted foot and bill actions (see Movie S1). This permitted the subject to insert and direct a stick tool despite the species’ morphological constraints. Kermit’s performance indicates a high degree of deliberate control over his movements, suggestive of anticipation of their effect and perhaps a representation of the goal action, i.e. of inserting the stick into the opening. Proof of goal directedness or goal representation, however, requires specific tests that were not implemented here [46].

Our study illustrates the difficulties of comparative cognition research and points to some partial solutions. Clearly, no single-task exploration can be used to assess problem-solving ability or make claims for advanced general intelligence or innovativeness. This caveat applies to within as well as between species comparisons. Problem solving is intrinsically multi-dimensional and it is to be expected that individuals or species will outperform each other in different dimensions. Batteries of tasks designed with this in mind may however be highly informative about the different predispositions and cognitive competences across individuals and species.

## Methods

### Subjects

Six male kea (Frowin, Kermit, Pick, Tammy, Bruce and Luke), as well as five New Caledonian crows (Boycott, Annie-Claude, Tino, Ebony and Uék), two of which were male (Boycott and Tino) participated in this study. Three kea (Kermit, Pick and Tammy) as well as one crow (Uek) were hand-raised. Bruce, Luke and Frowin were parent-raised in captivity; Annie-Claude, Tino, Ebony and Boycott were wild caught but had laboratory experience. Bruce and Luke were seven, Frowin, Pick and Kermit five, Tammy three and Uek, five years old. All subjects had experience in experimental problem-solving setups substantially different from that used here [8], [17], [18], [24], [30], [47], [48]. The NCC had participated in a test where they dropped stones into vertical tubes [24]. Kea also inserted objects into vertical tubes (unpublished data) and participated in follow up tool-use experiments with loose rewarded tubes or tubes in a slanted position and compact tools [18], [30]. Both the NCC and the kea had experiences with strings: the kea had participated in a vertical string-pulling task [32, and unpublished data] and the crows were given experience, pulling 30 cm long strings (of different colour and diameter as during testing) up a perch prior to a yet unpublished study. The crows had experience with stick tools within experimental and natural contexts.

The kea were housed in a large outdoor aviary (15 m ×10 m ×4 m) in a group totalling 20 kea, while the crows were kept in pairs in outdoor aviaries of various shapes, with an average volume of approximately 50 m^3^ and access to heated indoor divisions (ca. 8 m^3^), with food and drinking water *ad libitum*. The experiments were strictly non-invasive and based purely on behavioral tests. All subjects were housed in accordance with Austrian and German law.

### Apparatus

We designed and used a Multi-Access-Box (MAB) consisting of a cubic box (23 cm by side; for details and further dimensions see Figure 1, see additionally Figures S1, S2, S3, S4, S5; Movie S2) with four exchangeable transparent walls (the dimensions were adequate for both species). Each wall contained an opening that could be used to access a food reward presented in the centre of the box, either directly or by means of a tool. The food reward (half a peanut in its shell for the kea; a mealworm inside half a peanut shell for the NCC) was positioned on a vertical pole in the centre of the box, which was attached to a slanted platform. Once the food fell off the pole it rolled down the platform and out of the box.

There were four possible solutions (‘string’, ‘window’, ‘ball’ and ‘stick’) through which the out-of-reach food could be obtained, two of which (‘ball’ and ‘stick’) involved the use of tools. (i) The ’string’ solution required the subjects to pull a string (20 cm) hanging out of the opening in the respective wall, the other end of which was tied to the reward. (ii) In the ‘window’ option, a hinged window in the sidewall of the box could be opened by grasping a hook-like handle with the beak, pulling he window open and thereafter reaching into the box to retrieve the food from the pole (or pushing it off the pole with a stick). (iii) To exploit the ‘ball’ option, a compact object (a marble) had to be inserted into the respective opening, which connected to a transparent tube bending towards the central pole. When inserted into the tube the ball rolled down the chute and knocked the reward off the pole. (iv) Finally, the opening corresponding to the ‘stick’ solution was connected to a (8 mm) short straight horizontal tube at the same height as the food, but with a ten centimetre gap to the pole (Figure 1). Here, the food could be obtained by inserting a stick tool in the correct, hence unobstructed, opening, manoeuvring it towards the pole and hitting the peanut (kea) or stuffed peanut shell (NCCs).

All openings except for the window had the same round shape and diameter, hence were superficially perceptually similar, but could be distinguished by the visible internal structure (see Figure 1). Four sticks (15 cm long) and four balls, all painted with yellow childproof acrylic varnish, were provided in two different sizes. Two of the four supplied sticks were broad and two thin (0.5 cm and 1,5 cm in diameter respectively), whilst the balls were two large and two small marbles. The potential tools were placed in the four corners of the MAB so that each corner had one stick and one marble (the possible combinations of tool diameters were randomly assigned). The MAB was turned around before each trial and the walls were switched; so that the openings were at randomly changing positions.

### Experimental procedure

Prior to the start of the actual tests, the birds received at least four familiarization trials, in which each of the walls was missing once so the birds could just reach into the box and take the food reward. The crows, which are more neophobic than the kea, received as many familiarization trials as necessary to recover the reward in less than three minutes.

During testing, subjects were visually isolated from their group/mates and received a maximum of ten trials per session. A trial continued until the reward was recovered or until ten minutes had passed. If an animal did not obtain the reward within ten minutes testing continued the following day. A trial was scored as correct if the bird obtained the reward by applying one of the four solutions described above without prior unsuccessful attempts to solve the problem in a different way, e.g. previously manipulating other openings or combining the tools with wrong openings. The first successful retrieval of the food from a new opening (the bird may have manipulated other entrances in the same trial) was scored as a ‘discovery’.

Initially all openings were available. Once a subject reached criterion for one solution (obtaining the reward by always using the same solution for two consecutive sessions, but also after nine correct trials within one session (of ten trials) or eight consecutive correct trials using the same solution within one session), the respective opening was blocked (the window was cemented into its frame, the string was removed and the tool entrances were blocked with a wooden stopper), so as to force the subject to shift to other solutions. Testing continued until the animals failed to recover the reward within ten minutes in three consecutive trials or until all openings were closed. In order to determine whether each species had the capacity for each tool option, if just one of the two tool openings remained open, and the birds failed to solve it three consecutive times, we gave them a ‘second chance’ (another three trials) and removed the tools that belonged to the tool task they had previously solved, so as to remove possible distracting factors. We reasoned that the birds may fail to solve a tool task simply because they might not be able to inhibit using a tool which has been strongly associated to food before.

All data was videotaped. We used SPSS for statistical analysis.

## Supporting Information

Movie S1 This video shows the complex motor technique used by the male kea Kermit to insert the rod shaped tool into the appropriate opening as described in the results section.
(AVI)

Movie S2 This ‘Kea-crow suite movie’ shows both species, kea and New Caledonian crows, employing all four different solutions of the Multi Access Box apparatus: string, ball, stick and window. The video illustrates the techniques used to employ the fourth and final solution of the two animals, the kea Kermit and the crow Uek, that mastered all tasks: the crow Uek uses a tool to poke the reward off its platform after opening the window solution and the kea Kermit uses a complex multi step technique to insert the stick tool into the appropriate opening (as described in detail in the results section).
(M4V)

Figure S1 This image depicts a kea inserting a ball tool into the appropriate opening.
(JPG)

Figure S2 This image depicts a New Caledonian crow inserting a ball tool into the appropriate opening.
(JPG)

Figure S3 This image depicts the crow Uek retrieving the reward from the window opening using a stick tool.
(JPG)

Figure S4 This image depicts a kea opening the window solution.
(JPG)

Figure S5 This image depicts a kea inserting the stick tool into the appropriate opening.
(JPG)
