# Supplementary figures and images for: Flexibility in Problem Solving and Tool Use of Kea and New Caledonian Crows in a Multi Access Box Paradigm
Source: PLoS One. 2011 Jun 8;6(6):e20231. doi: 10.1371/journal.pone.0020231 (PMC3110758; doi:10.1371/journal.pone.0020231)

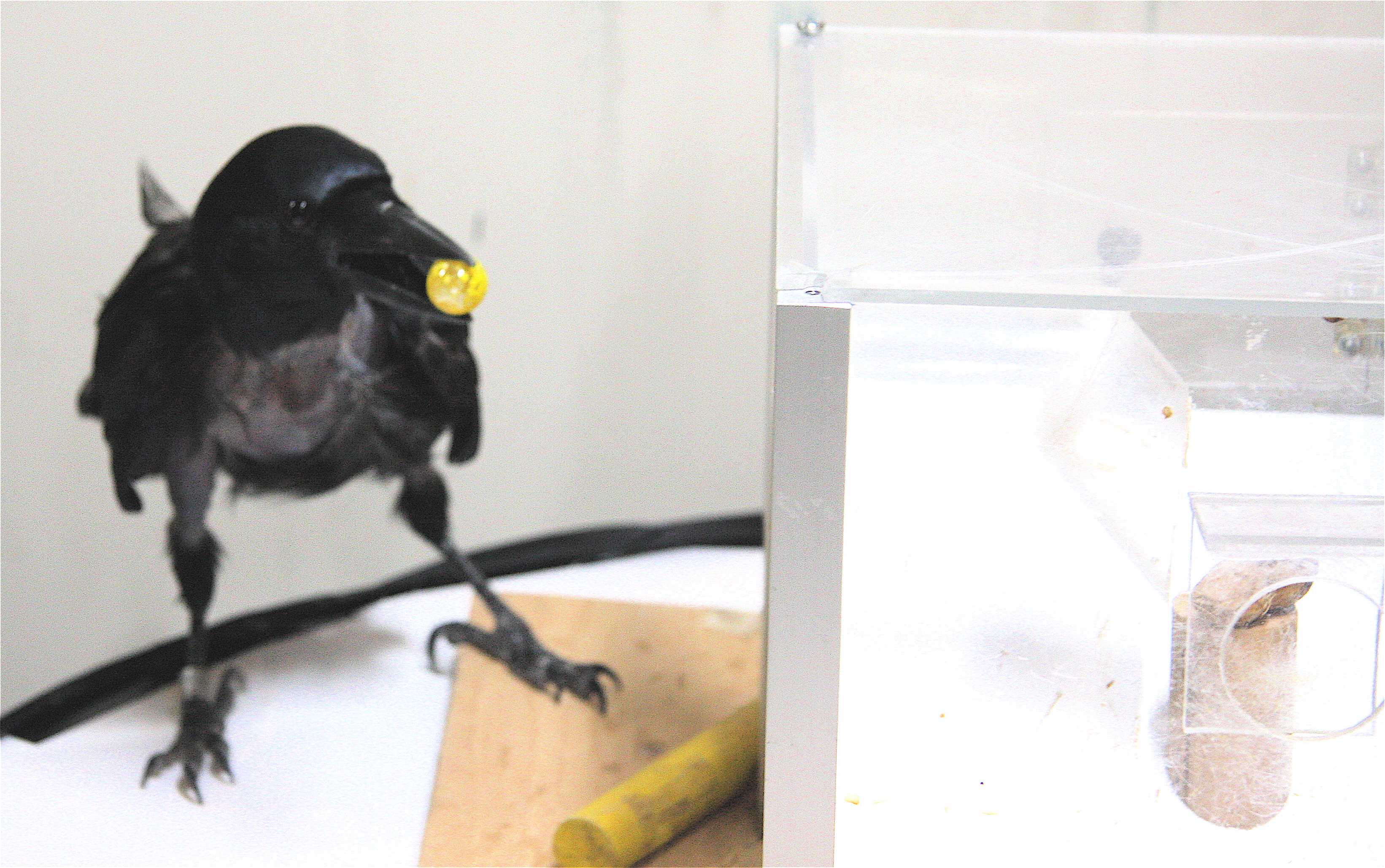

Supplement: Figure S1 — This image depicts a kea inserting a ball tool into the appropriate opening. (JPG) [file pone.0020231.s003.jpg]

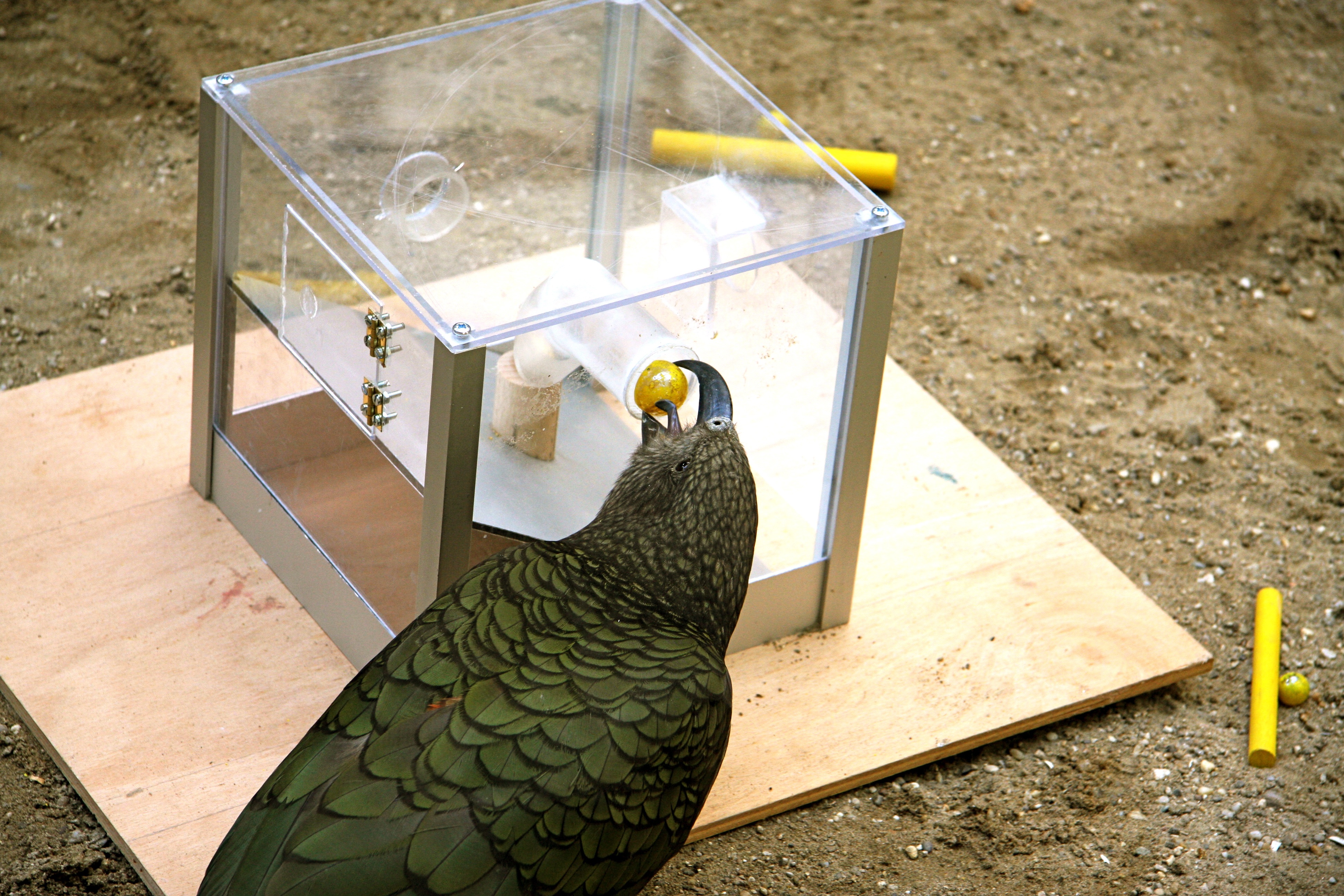

Supplement: Figure S2 — This image depicts a New Caledonian crow inserting a ball tool into the appropriate opening. (JPG) [file pone.0020231.s004.jpg]

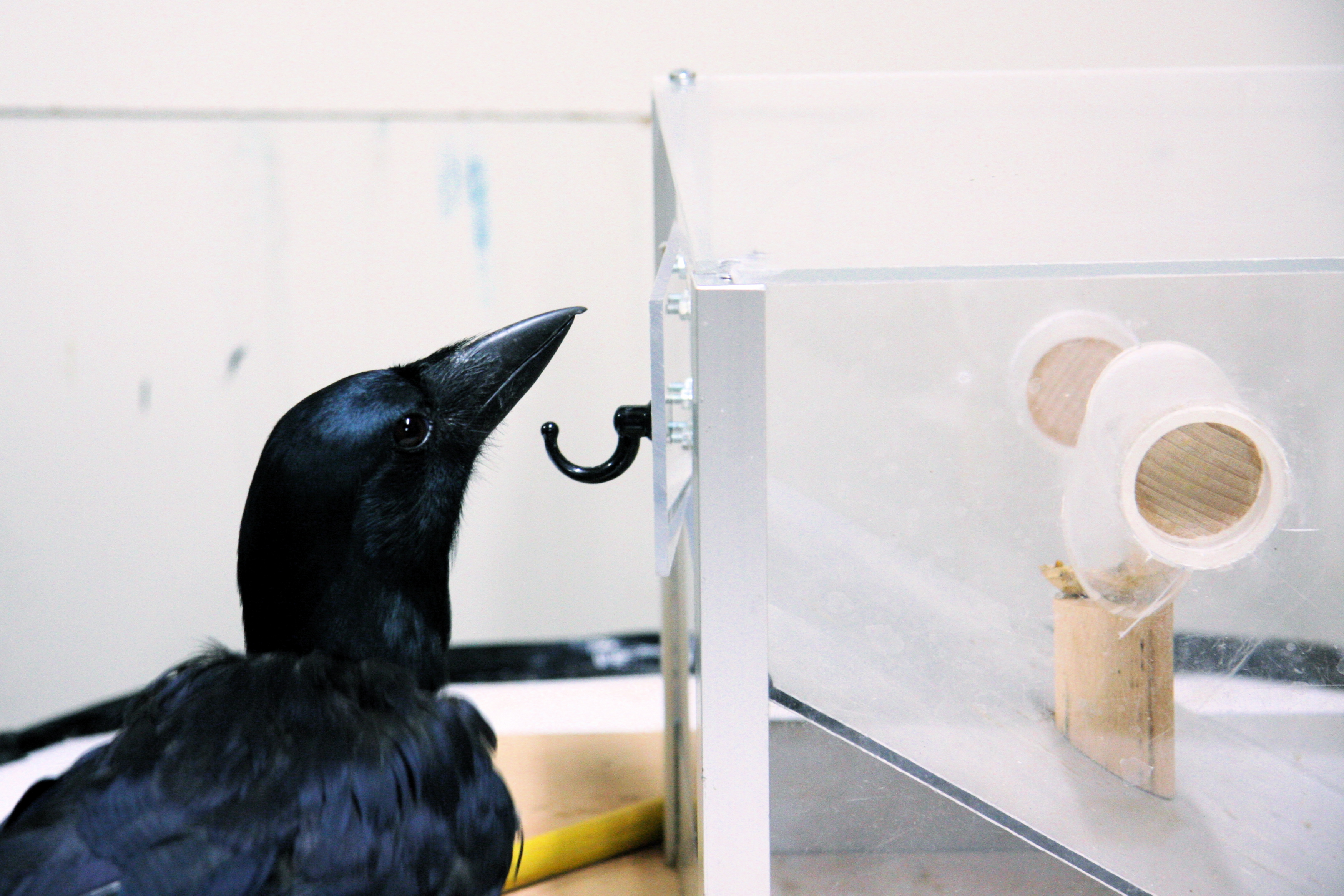

Supplement: Figure S3 — This image depicts the crow Uek retrieving the reward from the window opening using a stick tool. (JPG) [file pone.0020231.s005.jpg]

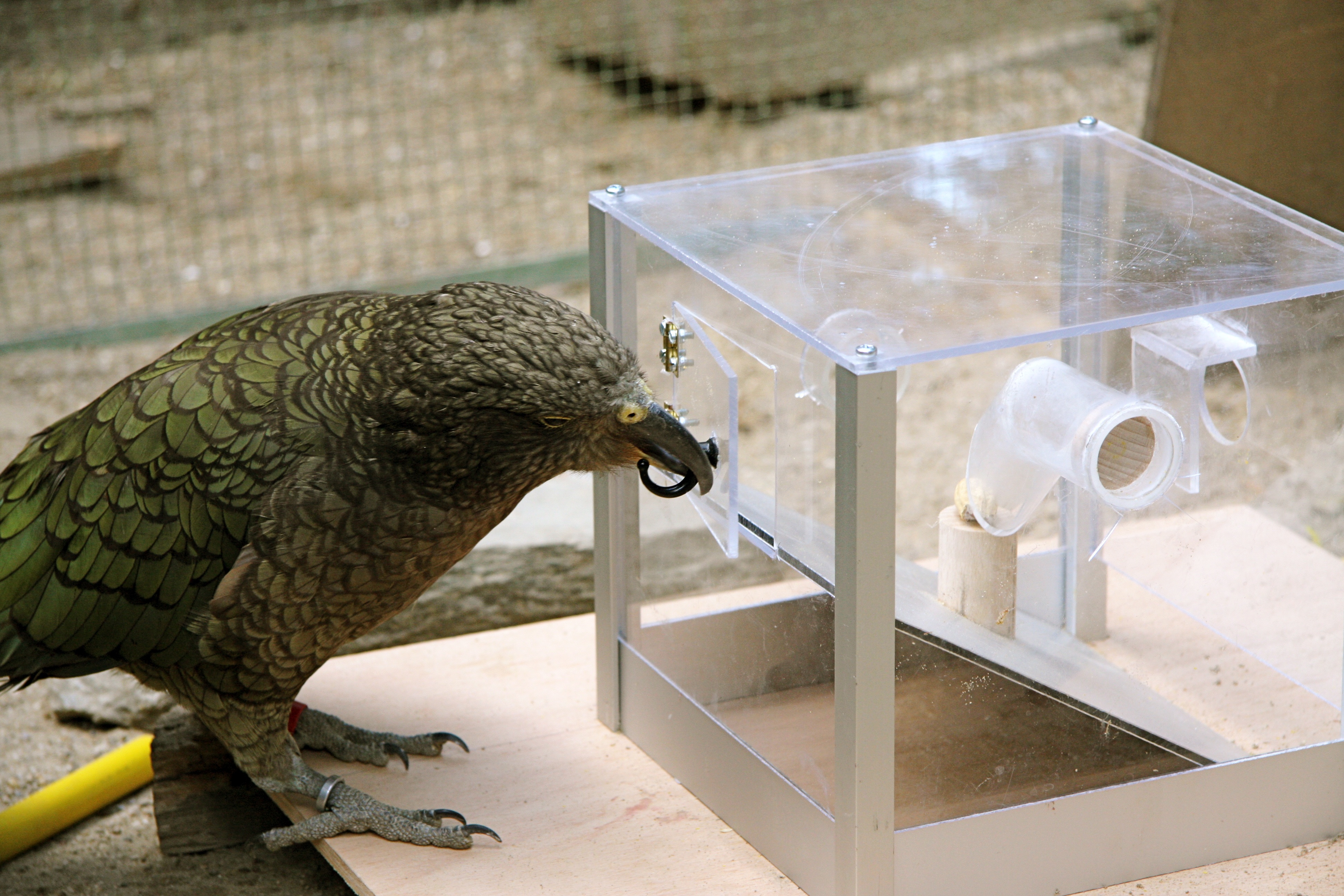

Supplement: Figure S4 — This image depicts a kea opening the window solution. (JPG) [file pone.0020231.s006.jpg]

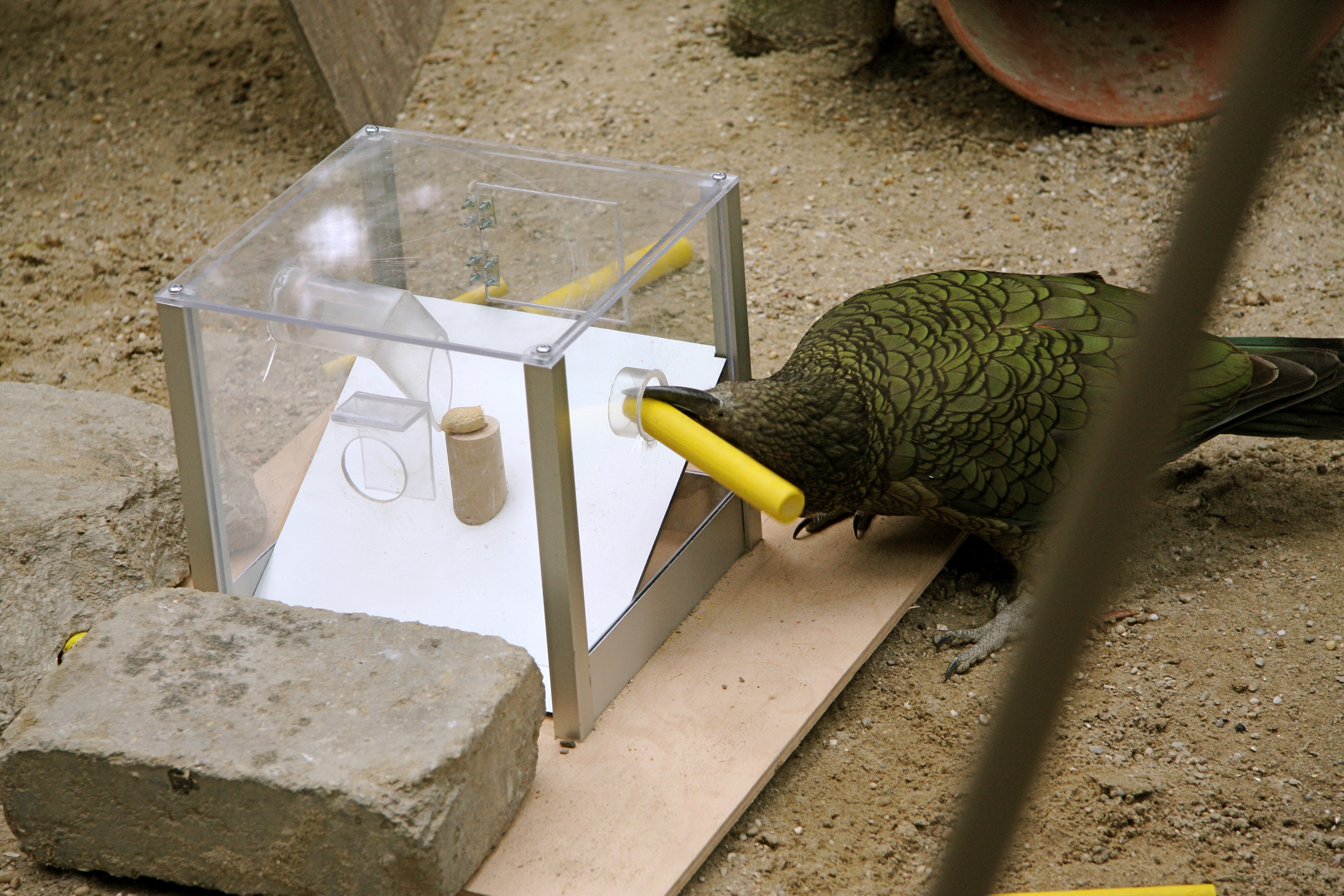

Supplement: Figure S5 — This image depicts a kea inserting the stick tool into the appropriate opening. (JPG) [file pone.0020231.s007.jpg]
